# Artificial Intelligence Approach for Classifying Images of Upper-Atmospheric Transient Luminous Events

**DOI:** 10.3390/s24103208

**Published:** 2024-05-18

**Authors:** Axi Aguilera, Vidya Manian

**Affiliations:** Department of Electrical and Computer Engineering, University of Puerto Rico at Mayaguez, Mayagüez, PR 00681, USA; axi.aguilera@upr.edu

**Keywords:** TLEs, sprite, halo, elve, gigantic jet, deep learning, CNNs, transfer learning

## Abstract

Transient Luminous Events (TLEs) are short-lived, upper-atmospheric optical phenomena associated with thunderstorms. Their rapid and random occurrence makes manual classification laborious and time-consuming. This study presents an effective approach to automating the classification of TLEs using state-of-the-art Convolutional Neural Networks (CNNs) and a Vision Transformer (ViT). The ViT architecture and four different CNN architectures, namely, ResNet50, ResNet18, GoogLeNet, and SqueezeNet, are employed and their performance is evaluated based on their accuracy and execution time. The models are trained on a dataset that was augmented using rotation, translation, and flipping techniques to increase its size and diversity. Additionally, the images are preprocessed using bilateral filtering to enhance their quality. The results show high classification accuracy across all models, with ResNet50 achieving the highest accuracy. However, a trade-off is observed between accuracy and execution time, which should be considered based on the specific requirements of the task. This study demonstrates the feasibility and effectiveness of using transfer learning and pre-trained CNNs for the automated classification of TLEs.

## 1. Introduction

Transient Luminous Events (TLEs) represent a captivating and relatively recently acknowledged phenomenon in the field of atmospheric and space sciences. First documented in detail in the late 1980s [1], TLEs are transient optical emissions that occur in the upper atmosphere and are typically associated with thunderstorm activity and lightning strikes. These phenomena manifest in a variety of forms, including sprites, elves, blue jets, and others, each exhibiting distinct characteristics in terms of altitude, duration, and physical structure.

The study of TLEs is not only of academic interest but also holds substantial significance for atmospheric physics, meteorology, and space weather forecasting. Understanding TLEs contributes to our knowledge of the electrical and chemical processes in the Earth’s mesosphere and lower ionosphere, offering insights into the complex interactions between the Earth’s atmosphere and the environment of space. Over the past three decades, they have become a significant subject of interest, leading to a wide range of literature and theories covering their phenomenology and morphology [2].

There are different classes of TLEs, such as sprites, elves, halos, and jets, among others. The so-called sprites were discovered in 1990 and reported to the global scientific community in [1]. They occur above thunderclouds due to lightning strikes that transfer significant charge from the upper cloud regions. These flashes can stretch from 40 to 80 km in height, far above clouds’ maximum altitude of 15 km, and they can be dozens of kilometers wide. Typically, the upper sections of a sprite emit a faint red light known as a halo, whereas its lower sections exhibit structured blue streamers. However, it is possible to observe halos without streamers and vice versa. The duration of sprites varies from a few to several tens of milliseconds, making them just noticeable to the naked eye. The widely accepted explanation for sprite formation was initially proposed by the VLF Group in a series of studies conducted between 1995 and 1997 [3,4]. Halos are typically initiated by a positive cloud-to-ground lightning strike, similarly to sprites, and halos can often be observed alongside sprites. They usually form at an altitude of about 80–85 km and often appear as red, oval-shaped clouds [5,6].

Elves were theorized by the Very-Low-Frequency (VLF) Research Group before they were actually observed for the first time [7]. These types of TLEs are characterized by rapidly expanding circles of red light that originate high above the clouds due to lightning strikes. They spread out rapidly at the ionosphere’s lower edge (between 80 and 90 km in altitude), seeming to move faster than light, and they can reach up to 300 km in diameter in less than a millisecond. Due to their extremely quick nature, elves are too fast to be seen by the human eye and challenging to capture with standard 30 fps video cameras [8].

Blue jets were first documented in 1994 [9]. They are partly ionized luminous cones that are mostly blue in color and travel at rates of around 100 km/s upward from the summit of thunderstorms, reaching an altitude of up to 40 km [10]. Gigantic jets are created in the middle of a storm cloud; these are exceptionally rare phenomena that can extend up to altitudes as high as 80 km. They begin as a very bright white lightning bolt that ascends from the top of the cloud, changing color from blue to red as it ascends. As blue jets, they are also linked to upward electrical discharges emanating from the tops of clouds [5,6].

In order to develop an AI method for TLE image classification, the image characteristics of TLEs have to be understood. Blue jets are characterized by their narrow, cone-shaped projections extending upwards from thunderstorm clouds, often displaying a distinct blue hue. Elves, on the other hand, present as rapidly expanding concentric rings of red light, originating high above thunderclouds and propagating outward at the ionosphere’s lower edge. Gigantic jets manifest as exceptionally rare phenomena, featuring bright white lightning bolts ascending from storm clouds and transitioning in color from blue to red as they reach extreme altitudes. Halos typically appear as elliptical clouds with a reddish tint, forming at intermediate altitudes and often accompanying other TLEs. Sprites exhibit large-scale red structures extending vertically above thunderstorm clouds, with intricate branching patterns resembling trees. Sprite–halo events combine characteristics of sprites and halos, displaying both the distinctive vertical structure of sprites and the elliptical shape of halos. Sprite–jellyfish events resemble large column sprites with a shape reminiscent of a jellyfish, adding further complexity to the visual repertoire of TLEs. These varied visual signatures serve as essential cues for automated classification algorithms aiming to discern between different types of TLEs.

Currently, there exists only one prior work on the classification of TLEs. In [11], the authors developed a Convolutional Neural Network (CNN) model named HYDRA for four classes of images, including sprites, elves, and jets. This research paper is the second attempt to compile a repository of these images, preprocess them, and label them. In this era of AI, annotating and labeling datasets is the prime goal. Hence, the main contributions of this paper are the following:

1. Development of a workflow to preprocess the large repository of TLE images collected worldwide;
2. Development of pre-trained AI models for the classification and labeling of the TLE images.

The rest of this paper is organized as follows: Section 2 presents the materials and methods for TLE classification, Section 3 presents the results, Section 4 presents a discussion on the results, and Section 5 presents the conclusions.

## 2. Materials and Methods

### 2.1. Convolutional Neural Networks

Deep learning, a subfield of machine learning, has undergone significant development since its introduction. Its profound impact on various fields, including image classification, natural language processing, and computer vision, is noteworthy. In particular, Convolutional Neural Networks (CNNs), a class of deep learning models, are particularly suited to image classification tasks due to their ability to automatically learn hierarchies of increasingly complex features from raw input data.

The roots of deep learning and CNNs can be traced back to the Perceptron model introduced in the 1950s [12], which served as the foundation for artificial neural networks. However, due to the limitations in computational power and data availability, progress in the field was relatively slow until the 1980s [13]. The development of the backpropagation algorithm [14] by Rumelhart, Hinton, and Williams in 1986 provided a viable method for training multi-layer neural networks, allowing them to learn complex patterns in data.

In the late 1990s and early 2000s, advancements in computational power and the availability of larger datasets enabled significant progress in the field of deep learning. It was during this time that CNNs were introduced by Yann LeCun and colleagues [15], initially used for digit recognition in the postal service. CNNs differ from other neural network architectures in their use of convolutional layers, which apply a series of filters to the input data. These filters can automatically learn to detect different features in the images, such as edges, corners, and textures, making them highly effective for image classification tasks.

In recent years, the capabilities of deep learning and CNNs have been further showcased in several high-profile challenges and competitions, such as the ImageNet Large Scale Visual Recognition Challenge [16]. In 2012, a deep CNN known as AlexNet dramatically outperformed all previous methods in the competition, marking a significant turning point in the field of image classification [17,18,19,20].

CNN models have been extensively utilized for image classification, achieving such a degree of accuracy in object recognition that the error rate is remarkably low, at times even surpassing human precision. In references [21,22], both LeNet-5 and VGG19 are employed in the design of an automated network for classifying various types of wood. Al-Qizwini et al. [23] propose a deep learning algorithm for autonomous driving using GoogLeNet. After comparing the performance of three of the top-performing CNNs for extracting road features and their efficacy for autonomous driving, their results demonstrate that GoogLeNet is the most precise model for this task. In [24], ResNet and GoogLeNet are analyzed for the detection of malicious software (viruses), with ResNet proving to be the network with the highest accuracy. Lastly, in [25], GoogLeNet is adapted to design a network for recognizing handwritten Chinese characters.

**Figure 1:**
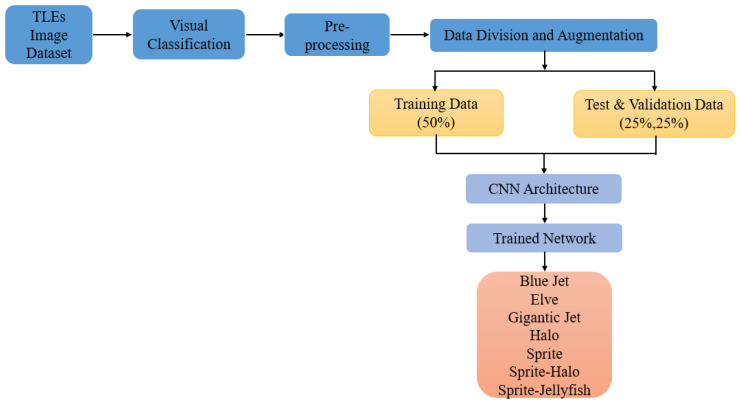
CNN architecture for TLE classification.

Applying deep learning and CNNs to the task of classifying Transient Luminous Events (TLEs) could be highly beneficial. The automatic feature extraction ability of CNNs could potentially allow them to learn to detect subtle patterns and characteristics in the images that may be difficult or time-consuming for human experts to identify manually. This could lead to more consistent and efficient classification of TLEs. Figure 1 shows a CNN architecture for TLE classification.

Moreover, the transfer learning approach allows us to leverage the vast amount of knowledge already learned by pre-trained models on large datasets such as ImageNet. By fine-tuning these models on the TLE dataset, we can benefit from the generic features learned by the models and adapt them to the specific task of TLE classification. Given the relatively small size of the TLE dataset, transfer learning can help mitigate the risk of overfitting and improve the generalizability of the model.

### 2.2. Vision Transformer

The Transformer architecture is a recursive-avoidant design that predominantly relies on an attention mechanism to establish comprehensive dependencies between input and output sequences. Prior to the advent of Transformers, prevailing models for sequence transduction relied on intricate recurrent or convolutional neural networks, comprising both an encoder and a decoder. While the Transformer architecture also encompasses an encoder and a decoder, its abandonment of recursion in favor of attention mechanisms enables considerably enhanced parallelization capabilities compared to methodologies such as Recurrent Neural Networks (RNNs) and CNNs [26].

**Figure 2:**
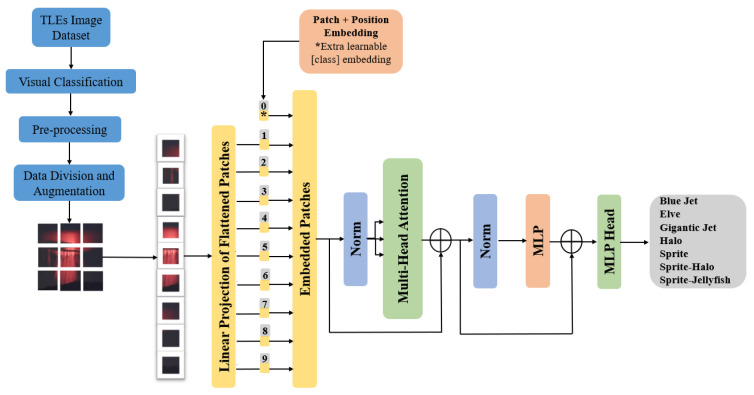
Architecture of the ViT for TLE classification based on [27].

Vision Transformer (ViT), introduced in [27], adapts the Transformer architecture for natural language processing to process images by dividing them into patches. The image is initially partitioned into fixed-sized patches, each of which undergoes linear embedding; positional embeddings are then added, and the resulting sequence of vectors is subsequently fed into a conventional Transformer encoder. To facilitate the classification task, the standard approach of incorporating an additional “classification token” is employed, which can be learned within the sequence; see Figure 2. This approach has demonstrated impressive performance on various image classification tasks and is known for its ability to capture both local and global image information effectively.

ViT offers a complementary approach to TLE classification by directly modeling the interactions between image patches, allowing them to capture global context and long-range dependencies in TLE imagery. While CNNs focus on local spatial features, ViTs attend to the relationships between image patches across the entire image, enabling them to capture holistic information about the scene. This is particularly beneficial for TLE classification, as it allows ViTs to consider the broader context of each image, including the relative positions and interactions of different TLE types within the scene. By leveraging self-attention mechanisms, ViTs can effectively integrate information from distant image regions, facilitating robust classification performance even in complex and cluttered scenes.

### 2.3. Transfer Learning Approach

Transfer learning (TL) has emerged as a powerful technique in the machine learning and deep learning landscape, particularly for problems with limited data availability or computational constraints. The principle underlying TL is the application of knowledge acquired from one problem to a similar but distinct problem, effectively leveraging prior learning to expedite the learning process on new tasks. This approach has shown significant promise in the realm of image classification, where models pre-trained on large datasets such as ImageNet have exhibited the ability to capture universal image features that prove beneficial across various tasks.

In the context of Transient Luminous Event (TLE) classification, the application of TL is not only apt but potentially advantageous due to several factors:

1. Limited data availability: The collection of TLE images for classification tasks is an inherently challenging process resulting in scarce data. Training a deep neural network from scratch under such conditions is often prone to overfitting. TL offers a solution by enabling the model to learn from a larger dataset and subsequently fine-tune the learned representations to the specific smaller TLE dataset, thereby preventing overfitting.
2. Computational efficiency: The process of training a deep neural network from scratch necessitates considerable computational resources and time, both of which can be conserved through the use of TL. The fine-tuning of a pre-trained model through TL can be accomplished relatively rapidly and with fewer computational resources.
3. High-level feature extraction: Models pre-trained on large datasets such as ImageNet are adept at extracting complex, high-level features from images. These features can be instrumental in classifying intricate phenomena, such as TLEs.

Historically, the success of TL is well documented across various domains. In medical imaging, where data scarcity is a common issue due to the difficulty of obtaining medical scans, TL has significantly improved model performance [28]. Similarly, in the field of natural disaster damage assessment, where data are relatively limited, TL has enhanced classification task accuracy [29].

### 2.4. Algorithms: The Transfer Learning Workflow

The process of transfer learning typically follows a sequence of steps, although the specifics can vary depending on the architecture and application in question.

- Firstly, a pre-trained model is chosen. For this work, we select four pre-trained models, for example, ResNet50, a 50-layer-deep model trained on classifying 1000 different objects.
- Subsequently, the final layers of the model are replaced. This allows for the retraining of the model on a new set of images and classes. The final fully connected layer is altered to have a number of nodes equal to the new class count, and a novel classification layer is introduced to generate output based on probabilities determined by the softmax layer.

After modification, the final fully connected layer will denote the new class count that the model will learn, while the classification layer will yield outputs from the newly available output categories. For instance, while ResNet50 was initially trained on 1000 categories, replacing the final layers allows it to be retrained on any desired number of object categories.

- There is also an option to freeze the weights. The learning rates in the earlier network layers can be set to zero, effectively freezing the weights. During training, the parameters of these frozen layers remain static, thereby expediting the network training. Freezing weights can also mitigate the overfitting of the model to the new dataset, particularly when the dataset is small.
- The model is then retrained, which enables it to learn and recognize features linked to the new images and categories. Generally, retraining necessitates less data than building a model from scratch.
- Lastly, following the retraining of the model, new images can be classified, and the network performance can be evaluated.

The CNNs ResNet18, ResNet50, GoogLeNet, and SqueezeNet have proven their efficacy across a wide range of image classification tasks, making them suitable candidates for transfer learning to classify Transient Luminous Events (TLEs). ResNet50, developed by He et al. [20], with a residual learning framework, is designed to train very deep networks by directly learning the residual functions with reference to the layer inputs, mitigating the vanishing gradient problem. These networks have shown exceptional performance in tasks requiring rich feature hierarchies, making them ideal for TLE classification.

## 3. Optimizers in Machine Learning Model Training

In the training of machine learning models, the choice of optimizer plays a crucial role in the convergence and final performance of the model. In this study, we opted to use two widely used optimizers in the machine learning community: ADAM (Adaptive Moment Estimation) and SGD (Stochastic Gradient Descent). Below is a theoretical and mathematical description of each of them, as well as a justification for their choice in the context of this research.

ADAM is an optimization algorithm that combines the ideas of SGD with learning rate adaptation techniques. It was proposed by Diederik P. Kingma and Jimmy Ba in their seminal 2014 paper [30]. ADAM computes an adaptive estimate of the first and second moments of the gradients and uses this information to update the model parameters. The parameter update is performed as follows:

$$\begin{array}{cc} m_{t} & =\beta_{1}m_{t-1}+\left(1-\beta_{1}\right)g_{t}\\ v_{t} & =\beta_{2}v_{t-1}+\left(1-\beta_{2}\right)g_{t}^{2}\\ {\hat{m}}_{t} & =\frac{m_{t}}{1-\beta_{1}^{t}}\\ {\hat{v}}_{t} & =\frac{v_{t}}{1-\beta_{2}^{t}}\\ \theta_{t+1} & =\theta_{t}-\alpha \frac{{\hat{m}}_{t}}{\sqrt{{\hat{v}}_{t}}+\epsilon } \end{array}$$

where $m_{t}$ and $v_{t}$ are the estimates of the first and second moments of the gradient, respectively, $\beta_{1}$ and $\beta_{2}$ are the momentum decay coefficients, α is the learning rate, and ϵ is a small term to avoid division by zero.

SGD is a classic optimization algorithm that uses the gradient of a data sample to update the model parameters. At each iteration, SGD computes the gradient of the loss function with respect to a batch of data and performs a parameter update in the opposite direction of the gradient. The parameter update is performed as follows:

$$\theta_{t+1}=\theta_{t}-\alpha \nabla f\left(\theta_{t}\right)$$

where $\theta_{t}$ is the parameter vector at iteration *t*, α is the learning rate, and $\nabla f(\theta_{t})$ is the gradient of the loss function with respect to $\theta_{t}$.

The choice of ADAM and SGD is based on their effectiveness and popularity in the machine learning community. ADAM is known for its ability to dynamically adapt the learning rate and maintain good performance even in problems with sparse or ill-conditioned gradients. On the other hand, SGD is a simple and widely used optimizer that can be effective in classification problems such as the present one.

### 3.1. Data

**Figure 3:**
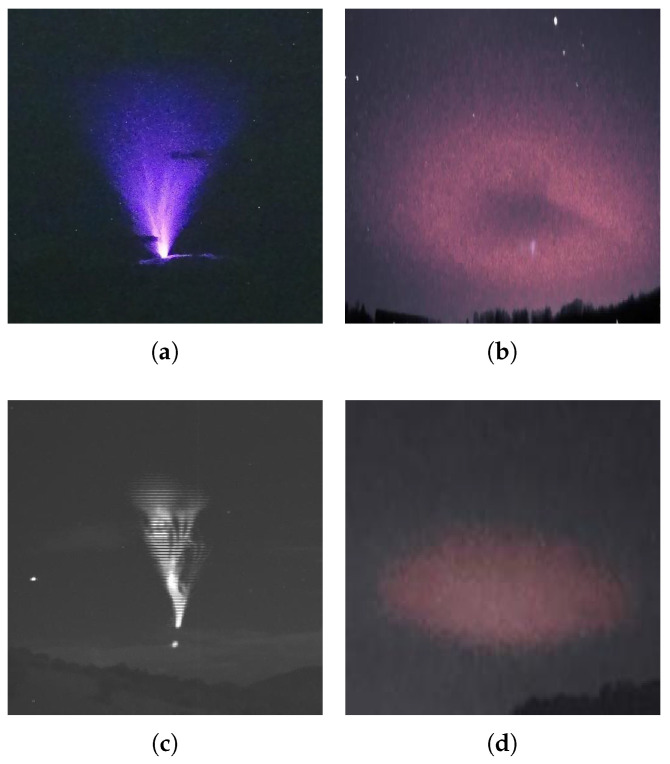
Sample of images in the collected data. (**a**) Blue jet; (**b**) elve; (**c**) gigantic jet; (**d**) halo.

**Figure 4:**
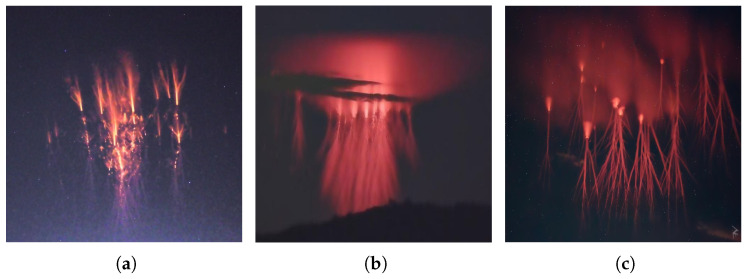
Sample of images of sprites in the collected data. (**a**) sprite; (**b**) sprite–halo; (**c**) sprite–jellyfish.

The image dataset used for training each CNN consists of photographs captured by TLE chasers around the world. A considerable portion of these images were generously provided by the Caribbean TLE observatory and were captured using cameras of various resolutions. Additionally, a significant number of images were sourced from the Facebook group “International Observers of Upper-Atmospheric Electric Phenomena”. Some images from the databases are displayed in Figure 3 and Figure 4. The images downloaded are from accurately labeled databases and have diverse images captured from different regions in Europe and the Americas. These are original images of TLEs captured when the events occurred in the upper atmosphere and, hence, are representative of each category of TLE.

The number of TLEs images per class is shown in Table 1; note that the numbers of images for the sprite, sprite–halo, and gigantic jet classes are much higher than the rest: 364, 50, and 49, respectively. Class imbalance can cause a machine learning model to be biased toward classes with more examples. In other words, the model can learn to favor classes with larger numbers because it sees more examples of them during training. As a result, the model may have a poorer performance in the classes with less data.

### 3.2. Preprocessing

Preprocessing of the TLE image dataset is a crucial step in the preparation of the data for the training of CNNs. This step ensures that the data fed into the models are of consistent quality and structure, enabling more accurate learning and subsequent predictions. Preprocessing not only includes tasks such as resizing and normalization but also involves the application of filters to enhance the quality of the images, especially in situations where the images may contain noise or other distortions [17].

Bilateral Filtering (BlF) [31] is an image processing technique used to enhance image quality while preserving edges and important details. This technique is particularly useful when working with noisy or low-contrast images, as it can help reduce noise without degrading edge quality. BlF is based on two main components: a spatial filter and a range filter. The basic idea is to smooth the image based on both the spatial proximity and intensity similarity between pixels.

The equation for BlF is defined as follows:

$$I_{\mathrm{bf}}\left(x\right)=\frac{1}{W_{p}}\sum_{x_{i}\in \Omega }I\left(x_{i}\right)\cdot G_{\mathrm{spatial}}(||x-x_{i}\left||\right)\cdot G_{\mathrm{range}}(|I\left(x\right)-I\left(x_{i}\right)\left|\right)$$

where

- $I_{\mathrm{bf}}$ is the filtered pixel value at position *x*.
- $I(x_{i})$ is the pixel value at position $x_{i}$, belonging to the neighborhood Ω of pixel *x*.
- $G_{\mathrm{spatial}}$ is a spatial weight function that measures the spatial proximity between *x* and $x_{i}$.
- $G_{\mathrm{range}}$ is a range weight function that measures the intensity similarity between I(x) and $I(x_{i})$.
- $W_{p}$ is a normalization factor that ensures that the sum of weights equals 1.

The functions $G_{\mathrm{spatial}}$ and $G_{\mathrm{range}}$ can be defined in various ways, but fundamentally, the closer *x* and $x_{i}$ are in terms of spatial distance and the more similar their intensities, the higher the weight they will receive in the calculation of the filtered value $I_{\mathrm{bf}}$.

These are some of the reasons why BlF is a suitable technique to improve the quality of TLE images and preserve their essential details:

1. Edge preservation: BlF preserves edges and important details in the image. This is crucial for our task, since TLEs may be associated with specific image features that must be retained.
2. Noise reduction: BlF can effectively reduce noise in images without excessively smoothing out details. This is beneficial, as our images are noisy.
3. Contrast enhancement: It can help enhance contrast in images, making relevant features more distinguishable and aiding in the classification task.
4. Tolerance to low resolution: BlF is robust to low resolution because it relies on spatial proximity and intensity similarity, which can be useful for dealing with low-quality images.

### 3.3. Performance Assessment Metrics

To evaluate the performance of the different models in classifying new images, a classic tool in machine learning evaluation is utilized: the confusion matrix. The experiments conducted in this research were assessed using standard metrics, such as recall, precision, F-score, specificity, and accuracy; these are defined in [32] as follows:

1. Precision indicates the proportion of samples correctly classified as positives among all samples classified as positives.
  $$Precision=\frac{TP}{TP+FP}$$
2. Specificity indicates the proportion of negative samples correctly classified among all true negative samples.
  $$Specificity=\frac{TN}{TN+FP}$$
3. Sensitivity (recall) represents the proportion of true positive samples that are correctly classified among all true positive samples.
  $$Recall=\frac{TP}{TP+FN}$$
4. F_1_ Score is the harmonic mean of precision and recall, providing a balance between both metrics.
  $$F_{1}\;Score=\frac{2\cdot Precision\cdot Recall}{Precision+Recall}$$
5. The error rate indicates the proportion of samples that are classified incorrectly relative to all samples.
  $$Error\;rate=\frac{FP+FN}{TP+TN+FP+FN}$$
6. Accuracy represents the proportion of samples that are classified correctly relative to all samples.
  $$Accuracy=\frac{TP+TN}{TP+TN+FP+FN}$$

Here, the parameters TP, TN, FP, and FN are carefully calculated from the confusion matrix. These are defined as follows:

- True positives (TPs):The number of positive samples correctly identified as positive by the model.
- True negatives (TNs): number of negative samples correctly identified as negative by the model.
- False positives (FPs): number of negative samples incorrectly classified as positive.
- False negatives (FNs): number of positive samples incorrectly classified as negative.

These metrics provide a comprehensive understanding of the models’ performance in classifying TLE image data.

## 4. Results and Discussions

In this section, we present the results of classifying images of TLEs using various architectures of Convolutional Neural Networks and the Vision Transformer.

To train and evaluate the aforementioned architectures, a series of transformations are applied to each class of images in the original dataset. These transformations include rotations, random crops, and brightness and contrast adjustments, among others, with the aim of increasing data variability and improving model generalization. After applying these transformations, 500 images are obtained for each class.

Subsequently, this new dataset is randomly divided into three sets: a training set representing 50% of the total images, a validation set representing 25%, and a test set also representing 25%. This division is made to ensure an unbiased evaluation of the model’s performance and to prevent overfitting during training.

The primary objective of this section is to analyze and discuss the performance of these architectures and optimizers in terms of accuracy, sensitivity, specificity, and other relevant metrics using the validation and test datasets. First, we present the results obtained by training the models from scratch, providing insights into their initial performance. Subsequently, we discuss the results obtained through transfer learning, where the models leverage pre-trained weights from models trained on large-scale datasets such as ImageNet. Through this dual approach, we aim to evaluate both the baseline performance of the models and their ability to leverage prior knowledge for improved classification accuracy.

The results presented here provide valuable insights into the ability of these architectures to effectively classify different classes of TLEs in new images, as well as their generalization capabilities.

Through this detailed analysis, we aim to provide a deeper understanding of the performance of different architectures and optimizers in the task of TLE classification, which may be useful for future research endeavors.

During the training of CNNs, the loss function plays a crucial role in measuring the disparity between the model’s predicted results and the ground-truth labels. In this study, we employed the cross-entropy loss function for its effectiveness in multi-class classification tasks. The cross-entropy loss is widely used in classification problems, as it penalizes incorrect classifications more heavily, leading to faster convergence during training.

### 4.1. Results When Training from Scratch

#### 4.1.1. Model Performance and Tuning

In Table 2, the fine-tuned parameters and the overall results obtained during the training process of different architectures using two classical optimizers, ADAM and SGD, are presented. The primary task is to classify images into a set of seven classes of TLEs, evaluating the accuracy of each architecture, the training time, and other relevant parameters.

The architectures evaluated in this study include ResNet50, ResNet18, GoogLeNet, SqueezeNet, and ViT. It is observed that ResNet50 and ResNet18 consistently demonstrate higher accuracy than that of the other architectures for both optimizers. This observation suggests that the depth of the network significantly impacts the model’s learning and generalization capability. Additionally, ViT exhibits considerably lower accuracy than that of CNN-based architectures, which may be attributed to differences in image feature representation and processing.

This study evaluates two common optimizers: ADAM and SGD. Overall, it is observed that ADAM tends to converge faster during training compared to SGD, as evidenced by the shorter training times recorded for all architectures. However, in terms of accuracy, no significant difference is observed between the two optimizers. This suggests that while ADAM may offer faster convergence, it does not necessarily guarantee superior accuracy compared to SGD.

Hyperparameters such as the maximum number of epochs, batch size, and initial learning rate are also evaluated in this study. It is observed that a higher number of training epochs tends to improve accuracy but also increases training time. Similarly, a larger batch size can accelerate the training process but may require more memory and computational resources.

**Figure 5:**
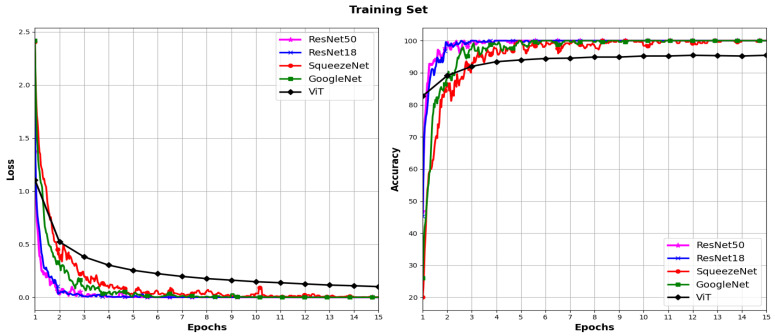

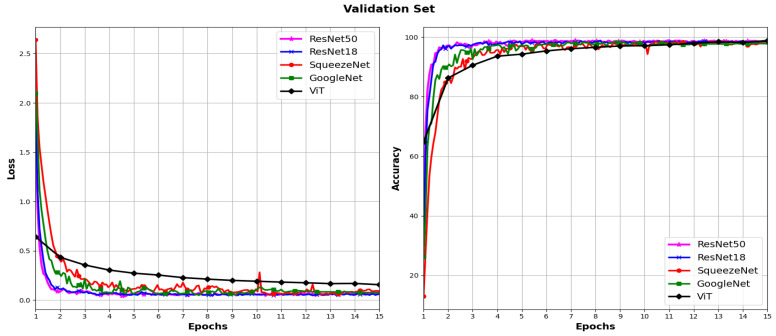
Loss and accuracy across different models using the ADAM optimizer.

**Figure 6:**
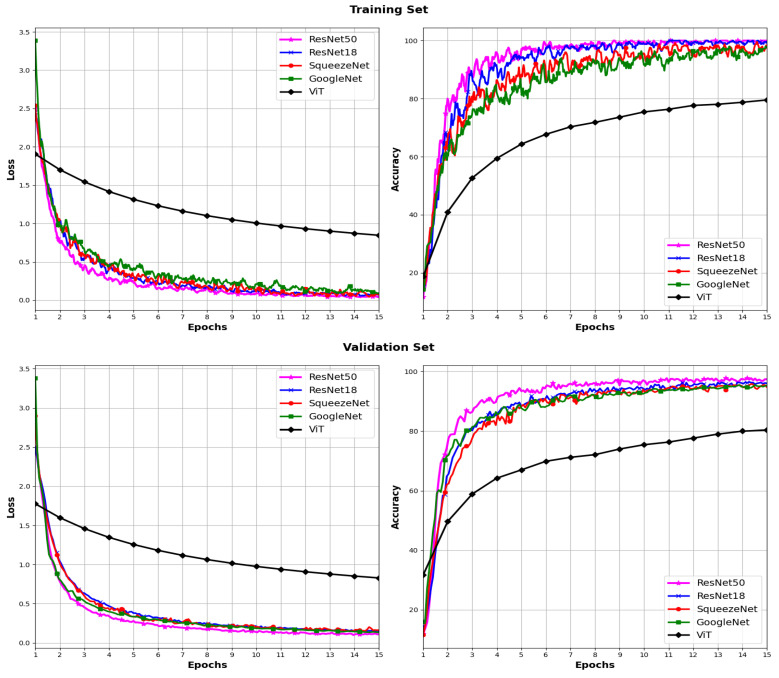
Loss and accuracy across different models using the SGD optimizer.

In Figure 5 and Figure 6, the loss and accuracy curves during the training and validation process for the five models using the ADAM and SGD optimizers, respectively, are plotted. For each model, a gradual decrease in loss is observed both in the training set and validation set across epochs. This decrease indicates effective learning as training progresses. However, it is noted that in the ViT model with the SGD optimizer, a significantly higher loss is observed compared to that of the rest of the architectures.

In terms of accuracy, a constant increase in accuracy is observed for both optimizers in both the training set and validation set for all models across epochs. This suggests that the models are improving their ability to correctly classify data as training progresses. The curves in Figure 6 show low accuracy for the ViT model in conjunction with the SGD optimizer, which is consistent with the results in Table 2. These results may indicate a slight overfitting to the training data.

#### 4.1.2. Accuracy Comparison per Class

The aim of this analysis is to examine the classification accuracy per class obtained using different architectures and optimizers. Table 3 provides a detailed overview of the classification accuracy for each class in the evaluated dataset.

The results indicate that the ResNet50 and ResNet18 architectures exhibit very high classification accuracy across all classes for both optimizers, ADAM and SGD. These architectures, renowned for their depth and ability to capture complex image features, demonstrate consistent and robust performance across most classes. Conversely, GoogLeNet, SqueezeNet, and ViT display variability in classification accuracy among classes, with some classes achieving close to 100% accuracy and others showing relatively lower accuracy. These differences can be attributed to variations in feature representation capabilities and architecture complexity.

Regarding the optimizers, the results do not show a significant difference in classification accuracy between ADAM and SGD for most classes across different architectures. However, there is a slight tendency towards higher accuracy with ADAM in some specific classes. This suggests that while the optimizer may influence the training process and model convergence, its impact on classification accuracy per class may be limited and largely dependent on other factors such as model architecture and dataset complexity.

Variability in classification accuracy per class is observed in the results, both among the different architectures and between the different optimizers. Some classes, such as “blue jet” and “gigantic jet”, exhibit close to 100% accuracy across all architectures and optimizers, while others, such as “sprite–halo” and “sprite–jellyfish”, demonstrate lower accuracy and greater variability among architectures and optimizers. This could be caused by similarities in geometry, luminosity, and colors between these event classes.

#### 4.1.3. Performance Evaluation of the Models on Testing Set

This section presents a detailed analysis of the performance of different machine learning models in classifying Transient Luminous Events (TLEs) using a previously unseen test set. The evaluated models include architectures based on CNNs such as ResNet50, as well as the novel ViT architecture.

The primary objective of this section is to provide a comprehensive and comparative evaluation of the models’ performance in the task of TLE classification. Key metrics such as accuracy, sensitivity, F1 score, and training time will be analyzed to assess the models’ ability to distinguish among different classes of TLEs and their computational efficiency.

#### 4.1.4. ResNet50’s Performance

**Figure 7:**
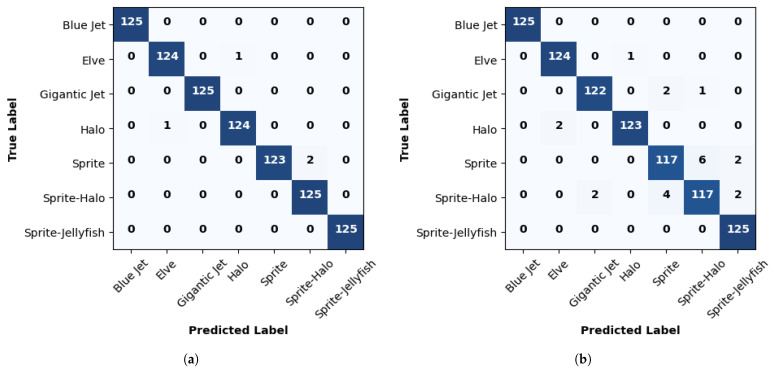
Confusion matrix assessment values for ResNet50. (**a**) ADAM optimizer; (**b**) SGD optimizer.

Table 4 provides a detailed insight into the performance of ResNet50 in classifying different classes of TLEs using two different optimizers: ADAM and SGD. The metrics defined in Section 3.3 were evaluated based on the confusion matrices in Figure 7 to better understand the model’s behavior in the classification task.

In terms of precision, it is observed that ResNet50 achieves a precision exceeding 98% in most classes when using the ADAM optimizer, indicating exceptional capability in correctly classifying different classes of TLEs. However, a slight decrease in precision is observed when utilizing the SGD optimizer, with the overall precision still being very high but slightly lower than that obtained with ADAM.

The sensitivity shows robust results, with values above 96% for most classes with both optimizers. This indicates that ResNet50 is highly sensitive in detecting the presence of TLEs in the input images. The F1 score, which combines precision and sensitivity into a single metric, shows values close to 100% in most classes, indicating a balance between precision and sensitivity. This suggests that the model achieves a good balance between the ability to correctly identify positive instances and avoid false positives.

#### 4.1.5. ViT’s Performance

This study presents a comprehensive evaluation of the ViT architecture’s performance in classifying TLEs using the ADAM and SGD optimizers. This analysis offers an in-depth understanding of the ViT’s ability to discern between different TLE classes and compares its performance with that of CNN-based models such as ResNet50.

**Figure 8:**
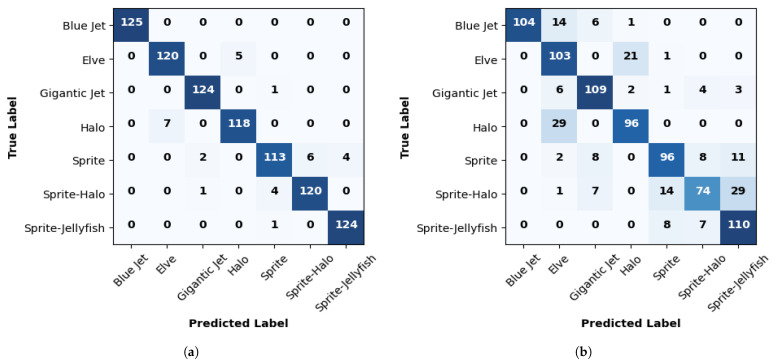
Confusion matrix assessment values for the Vision Transformer. (**a**) ADAM optimizer; (**b**) SGD optimizer.

Table 5 summarizes the results obtained from the confusion matrices in Figure 8, where it is observed that the ViT achieves comparable performance to that of ResNet50 in terms of metrics such as precision, sensitivity, and F1 score for most TLE classes. However, a decrease in performance is noted for specific classes, such as blue jets and elves, where the ViT exhibits a lower TP rate and a higher FN rate than those of ResNet50. This suggests that while the ViT effectively classifies TLEs overall, there may be areas for improvement in detecting certain specific classes.

Furthermore, Table 5 also reveals a discrepancy in the ViT’s performance when utilizing different optimizers. While the ViT with ADAM demonstrates more consistent results in terms of the precision and F1 score, the ViT with SGD displays a decline in these metrics, particularly in classes with fewer TPs. This implies that the choice of optimizer may influence the overall model performance, with ADAM potentially being more suitable for this specific classification problem.

Despite some performance variations between classes and optimizers, the ViT demonstrates robust generalization capabilities in TLE classification, with high precision and F1 score values across the majority of the evaluated classes. This indicates that the model can effectively learn relevant patterns from TLE images and apply this knowledge to accurately classify new instances.

### 4.2. Results from Transfer Learning

#### 4.2.1. Performance and Accuracy of Pre-Trained CNN Models

The aim of this subsection is to analyze the performance of pre-trained CNN models selected for the task of classifying TLE images. In Table 6, interesting patterns are observed in terms of classification accuracy and overall accuracy for the ResNet50, ResNet18, GoogLeNet, and SqueezeNet architectures.

ResNet50 and ResNet18 demonstrate similar overall accuracy in classifying TLE images, with values of 98.86% for ADAM and 97.60% for SGD in ResNet50 and 98.86% for ADAM and 96.57% for SGD in ResNet18. This suggests that although ResNet18 is a lighter version of ResNet50, both are equally effective in this task.

The GoogLeNet architecture exhibits slightly lower overall accuracy than that of ResNet50 and ResNet18, with values of 97.94% for ADAM and 94.74% for SGD. Conversely, SqueezeNet also demonstrates an overall accuracy comparable with that of ResNet18, with values of 98.06% for ADAM and 96.34% for SGD. Despite being lighter architectures, GoogLeNet and SqueezeNet demonstrate solid capabilities for TLE classification.

Upon examining the classification accuracy of different pre-trained architectures on ImageNet, it is observed that all exhibit strong performance across most TLE image classes. However, there are notable variations in classification accuracy among the architectures.

In the “blue jet” and “sprite–jellyfish” classes, all architectures achieve close to 100% accuracy with ADAM, indicating perfect classification for these specific classes. Conversely, the “sprite” and “sprite–halo” classes exhibit slightly lower accuracy than that of other classes for most architectures. This may be attributed to visual similarity between these classes and other categories, making precise distinctions challenging. GoogLeNet and SqueezeNet demonstrate slightly lower accuracy than that of ResNet50 and ResNet18 for most classes. Although these architectures have lower accuracy, their ability to classify TLE images remains considerably high.

An important consideration alongside accuracy is execution time. Both GoogLeNet and SqueezeNet are known for their computational efficiency and lower complexity; in the task of classifying TLE images, they have achieved competitive results in terms of accuracy with significantly shorter execution times than those of ResNet50 and ResNet18.

**Figure 9:**
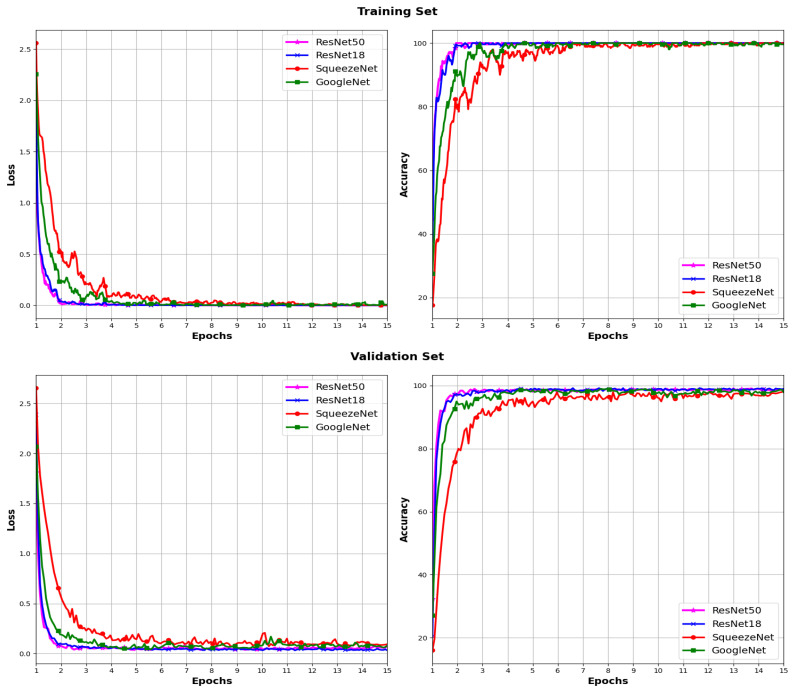
Loss and accuracy across different pre-trained CNN models using the ADAM optimizer.

**Figure 10:**
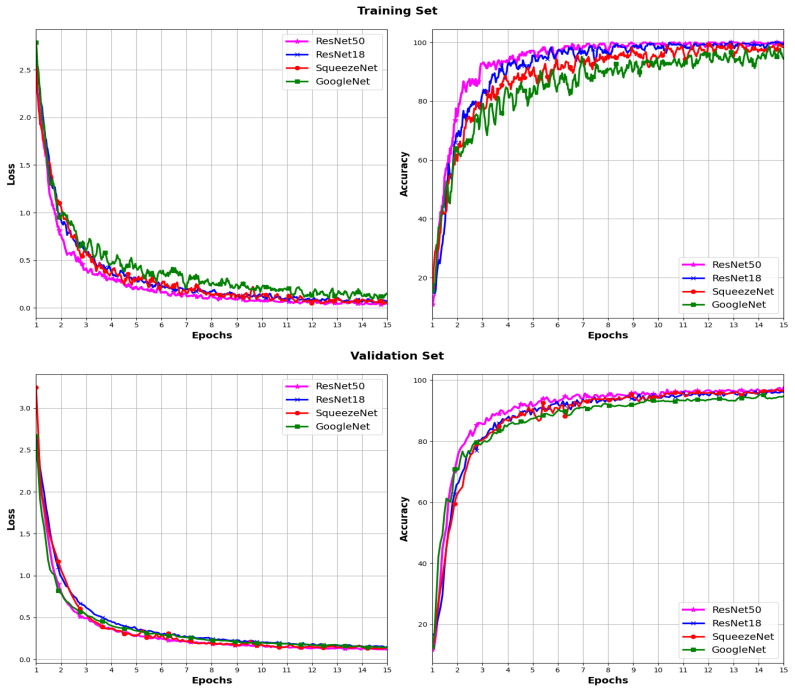
Loss and accuracy across different pre-trained CNN models using the SGD optimizer.

In Figure 9 and Figure 10, the loss and accuracy curves during the training and validation process for the four pre-trained CNN models using the ADAM and SGD optimizers, respectively, are depicted. Analogously to the models trained from scratch, these curves depict a consistent augmentation in accuracy across epochs for both the training and validation sets with each optimizer. Furthermore, there is an evident gradual decline in the loss for both the training and validation sets.

#### 4.2.2. Pre-Trained ResNet50’s Performance on the Testing Set

In this analysis, we assess the performance of the pre-trained CNN ResNet50 applied to the task of classifying TLE images. To comprehensively understand its classification capability, we examine various performance metrics, as presented in Section 3.3.

**Figure 11:**
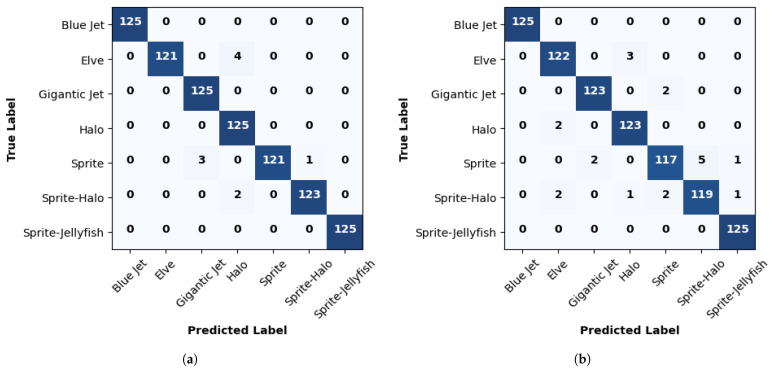
Confusion matrix assessment values for ResNet50 using transfer learning. (**a**) ADAM optimizer; (**b**) SGD optimizer.

The results in Table 7 demonstrate that the pre-trained ResNet50 exhibits outstanding performance in classifying TLE image classes. Figure 11 gives the confusion matrices for pre-trained ResNet50. Overall, it achieves 100% precision and recall for the “blue jet” class. Furthermore, it attains impressive results for the “gigantic jet” and “sprite–jellyfish” classes, with the precision and recall exceeding 99.5%. However, a slight decrease in precision and recall is observed for the “elve” and “sprite–halo” classes when the SGD optimizer is used compared to that when using ADAM.

In terms of precision and specificity, the pre-trained ResNet50 showcases notable results, with values surpassing 95% for most classes. The F1 score, which combines precision and recall, achieves values exceeding 98%, indicating good overall classification capability. On the other hand, the error rate is minimal, with values below 1.5% for all classes and optimizers.

## 5. Conclusions

The presented results demonstrate that the ResNet50, ResNet, GoogLeNet, SqueezeNet, and ViT architectures exhibit high performance in classifying the seven different classes of TLEs. The CNN architectures achieved precision rates exceeding 98% in most classes, indicating a significant ability to distinguish among different types of TLEs.

Regarding the utilized optimizers, both ADAM and SGD exhibited comparable results for most of the evaluated metrics. Nonetheless, for certain specific classes, such as blue jets, sprites, and gigantic jets, the ADAM optimizer exhibited a slight performance advantage over SGD in terms of precision and sensitivity.

When comparing the loss and accuracy curves among the five models, it is observed that all exhibit similar performance in terms of convergence and ability to learn from training data. However, the ViT model appears to have slightly inferior performance in terms of accuracy compared to the other models.

Notably, our experiments revealed that training the models from scratch and utilizing transfer learning yielded similar accuracy rates. However, it is important to highlight the considerable difference in computational costs between these two approaches. Training models from scratch requires more computational resources and time than using pre-trained models, whereas transfer learning significantly reduces the training time and resource requirements by leveraging pre-trained weights.

The findings outlined herein carry substantial implications for the advancement of automated TLE classification systems. This analysis is expected to help identify strengths and areas for improvement in the evaluated models, which, in turn, can guide future research efforts to further optimize the performance of TLE classification systems.

## Figures and Tables

**Table 1:** Number of TLE images per class.

TLE Class	Number of Images
Blue Jet	6
Elf	30
Gigantic Jet	49
Halo	9
Sprite	364
Sprite–Halo	50
Sprite–Jellyfish	21

**Table 2:** Comparison of the classification accuracy results (in percentage) and fine-tuned parameters for different architectures and optimizers.

Architecture	ResNet50		ResNet18		GoogLeNet		SqueezeNet		ViT	
Optimizer	ADAM	SGD	ADAM	SGD	ADAM	SGD	ADAM	SGD	ADAM	SGD
Accuracy	98.74	97.83	98.29	97.37	97.83	95.35	97.71	94.74	96.45	79.08
Maximum Epochs	15	15	15	15	15	15	15	15	15	15
Batch Size	50	50	50	50	50	50	50	50	50	50
InitialLearnRate	1.00 × 10^−4^	1.00 × 10^−4^	1.00 × 10^−4^	1.00 × 10^−4^	1.00 × 10^−4^	1.00 × 10^−4^	1.00 × 10^−4^	1.00 × 10^−4^	1.00 × 10^−4^	1.00 × 10^−4^
Training time (min/s)	603/53	569/7	357/42	250/3	380/58	244/57	170/46	171/1		

**Table 3:** Comparison of the classification accuracy (in percentage) per class for different architectures and optimizers.

Architecture	ResNet50		ResNet18		GoogLeNet		SqueezeNet		ViT	
Optimizer	ADAM	SGD	ADAM	SGD	ADAM	SGD	ADAM	SGD	ADAM	SGD
Blue Jet	100.0	100.0	100.0	99.77	100.0	99.89	99.89	99.54	100.0	97.60
Elve	99.77	99.66	99.77	99.77	99.66	98.63	99.43	98.51	98.63	91.54
Gigantic Jet	100.0	99.43	99.77	99.31	99.66	98.74	99.77	99.09	99.54	95.77
Halo	99.77	99.66	99.77	99.89	100.0	99.43	99.54	98.74	98.63	93.94
Sprite	100.0	99.54	100.0	99.54	99.89	98.74	99.77	98.74	99.43	93.37
Sprite–Halo	99.77	98.29	99.77	94.74	98.97	97.14	98.74	97.71	98.74	92.00
Sprite–Jellyfish	99.77	98.40	99.77	98.17	98.86	98.06	98.74	97.60	97.94	93.94

**Table 4:** Comparison of the classification results (in percentage) per class using ResNet50.

TLE Class	Blue Jet		Elve		Gigantic Jet		Halo		Sprite		Sprite–Halo		Sprite–Jellyfish	
Optimizer	ADAM	SGD	ADAM	SGD	ADAM	SGD	ADAM	SGD	ADAM	SGD	ADAM	SGD	ADAM	SGD
TP	125	125	124	124	125	122	124	123	123	117	125	117	125	125
TN	750	750	749	748	750	748	749	749	750	744	748	743	750	746
FP	0	0	1	2	0	2	1	1	0	6	2	7	0	4
FN	0	0	1	1	0	3	1	2	2	8	0	8	0	0
Specificity	100.0	100.0	99.87	99.73	100.0	99.73	99.87	99.87	100.0	99.20	99.73	99.07	100.0	99.47
Recall	100.0	100.0	99.20	99.20	100.0	97.60	99.20	98.40	98.40	93.60	100.0	93.60	100.0	100.0
Precision	100.0	100.0	99.20	98.41	100.0	98.39	99.20	99.19	100.0	95.12	98.43	94.35	100.0	96.90
F_1_ score	100.0	100.0	99.53	99.07	100.0	99.06	99.53	99.53	100.0	97.12	99.07	96.65	100.0	98.17
Error rate	0.00	0.00	0.23	0.34	0.00	0.57	0.23	0.34	0.23	1.60	0.23	1.71	0.00	0.46
Accuracy	100.0	100.0	99.77	99.66	100.0	99.43	99.77	99.66	99.77	98.40	99.77	98.29	100.0	99.54

**Table 5:** Comparison of the classification results (in percentage) per class using the Vision Transformer.

TLE Class	Blue Jet		Elve		Gigantic Jet		Halo		Sprite		Sprite–Halo		Sprite–Jellyfish	
Optimizer	ADAM	SGD	ADAM	SGD	ADAM	SGD	ADAM	SGD	ADAM	SGD	ADAM	SGD	ADAM	SGD
TP	125	104	120	103	124	109	118	96	113	96	120	74	124	110
TN	750	750	743	698	747	729	745	726	744	726	744	731	746	710
FP	0	0	7	52	3	21	5	24	6	24	6	19	4	43
FN	0	21	5	22	1	16	7	29	12	29	5	51	1	15
Specificity	100.0	100.0	99.07	93.07	99.60	97.20	99.33	96.80	99.20	96.80	99.20	97.47	99.47	94.00
Recall	100.0	83.00	96.00	82.40	99.20	87.20	94.40	76.80	90.40	76.80	96.00	59.20	99.20	88.00
Precision	100.0	100.0	94.49	66.45	97.64	83.84	95.93	80.00	94.96	80.00	95.24	79.57	96.88	72.00
F_1_ score	100.0	100.0	96.72	77.54	98.61	90.03	97.60	87.60	97.03	87.60	97.18	87.61	98.15	82.00
Error rate	0.00	2.00	1.37	8.46	0.46	4.23	1.37	6.06	2.06	6.06	1.26	8.00	0.57	7.00
Accuracy	100.0	98.00	98.63	91.54	99.54	95.77	98.63	93.94	97.94	93.94	98.74	92.00	99.43	93.00

**Table 6:** Comparison of the classification accuracy (in percentage) per class for different pre-trained CNN models and optimizers.

Architecture	ResNet50		ResNet18		GoogLeNet		SqueezeNet	
Optimizer	ADAM	SGD	ADAM	SGD	ADAM	SGD	ADAM	SGD
Blue Jet	100.0	100.0	100.0	100.0	100.0	99.66	100.0	99.54
Elve	99.54	99.20	100.0	100.0	99.77	98.63	99.66	98.51
Gigantic Jet	99.66	99.54	99.54	99.54	99.66	99.31	99.54	99.09
Halo	99.31	99.31	99.77	99.77	99.54	98.63	99.43	98.74
Sprite	99.54	98.63	99.09	99.09	99.09	97.94	99.09	97.60
Sprite–Halo	99.66	98.74	99.54	99.54	98.97	97.37	98.74	99.77
Sprite–Jellyfish	100.0	99.77	99.77	99.77	98.86	97.94	99.66	98.74
**General Accuracy**	98.86	97.60	98.86	96.57	97.94	94.74	98.06	96.34

**Table 7:** Comparison of the classification results per class for ResNet50 using transfer learning.

TLE Class	Blue Jet		Elve		Gigantic Jet		Halo		Sprite		Sprite–Halo		Sprite–Jellyfish	
Optimizer	ADAM	SGD	ADAM	SGD	ADAM	SGD	ADAM	SGD	ADAM	SGD	ADAM	SGD	ADAM	SGD
TP	125	125	121	122	125	123	125	123	121	117	123	119	125	125
TN	750	750	750	746	747	748	744	746	750	746	749	745	750	748
FP	0	0	0	4	3	2	6	4	0	4	1	5	0	2
FN	0	0	4	3	0	2	0	2	4	8	2	6	0	0
Specificity	100.0	100.0	96.80	97.60	100.0	98.40	100.0	98.40	96.80	93.60	98.40	95.20	100.0	100.00
Recall	100.0	100.0	100.0	99.47	99.60	99.73	92.20	99.47	100.0	99.47	99.87	99.33	100.0	99.73
Precision	100.0	100.0	100.0	96.83	97.66	98.40	95.42	96.85	100.0	96.69	99.19	95.97	100.0	98.43
F_1_ score	100.0	100.0	100.0	98.13	98.62	99.06	97.27	98.14	100.0	98.06	95.53	97.62	100.0	99.07
Error rate	0.00	0.00	0.46	0.80	0.34	0.46	0.66	0.69	0.46	1.37	0.34	1.36	0.00	0.23
Accuracy	100.0	100.0	99.54	99.20	99.66	99.54	99.31	99.31	99.54	98.63	99.66	98.74	100.0	99.77

## Data Availability

The data used in this paper is available from website: spritacular.org.
